# Corrigendum: Complex Dynamics in Simplified Neuronal Models: Reproducing Golgi Cell Electroresponsiveness

**DOI:** 10.3389/fninf.2019.00050

**Published:** 2019-07-10

**Authors:** Alice Geminiani, Claudia Casellato, Francesca Locatelli, Francesca Prestori, Alessandra Pedrocchi, Egidio D'Angelo

**Affiliations:** ^1^NEARLab, Department of Electronics, Information and Bioengineering, Politecnico di Milano, Milan, Italy; ^2^Department of Brain and Behavioral Sciences, University of Pavia, Pavia, Italy; ^3^IRCCS Mondino Foundation, Pavia, Italy

**Keywords:** neuronal modeling, point neuron, leaky integrate-and-fire, model simplification, neuronal electroresponsiveness, Golgi cell, cerebellum

In the published article, there was an error regarding the affiliation for “Egidio D'Angelo.” As well as having affiliation(s) “2,” they should also have “IRCCS Mondino Foundation, Pavia, Italy.”

The authors apologize for this error and state that this does not change the scientific conclusions of the article in any way. The original article has been updated.

