# Supplementary material for: Complex Dynamics in Simplified Neuronal Models: Reproducing Golgi Cell Electroresponsiveness
Source: Front Neuroinform. 2018 Dec 3;12:88. doi: 10.3389/fninf.2018.00088 (PMC6287018; doi:10.3389/fninf.2018.00088)
Supplement: Supplementary file 1 [file Data_Sheet_1.docx]

# Appendix

## E-GLIF model solution and parameter space

Considering the matrix form of the ODE system describing the model dynamics (Equ. 1):

$\frac{d}{dt}\left[ \begin{aligned} V_{m}(t) \\ I_{adap}(t) \\ I_{dep}(t) \end{aligned} \right]= \left[ \begin{matrix} \frac{1}{\tau_{m}} & -\frac{1}{C_{m}} & \frac{1}{C_{m}} \\ k_{adap} & {-k}_{2} & 0 \\ 0 & 0 & {-k}_{1} \end{matrix} \right]\cdot\left[ \begin{aligned} V_{m}(t) \\ I_{adap}(t) \\ I_{dep}(t) \end{aligned} \right]+\left[ \begin{aligned} -\frac{1}{\tau_{m}}E_{L}+\frac{1}{C_{m}}(I_{e}{+ I}_{stim}) \\ -k_{adap}\cdot E_{L} \\ 0 \end{aligned} \right]$ (A 1)

the general solution is:

$\left[ \begin{aligned} V_{m}(t) \\ I_{adap}(t) \\ I_{dep}(t) \end{aligned} \right]=c_{1}\cdot\boldsymbol{x}_{\boldsymbol{1}}\cdot e^{\lambda_{1}t}+c_{2}{\cdot\boldsymbol{x}_{\boldsymbol{2}}\cdot e}^{\lambda_{2}t}+c_{3}\cdot\boldsymbol{x}_{\boldsymbol{3}}\cdot e^{\lambda_{3}t}+\left[ \begin{aligned} V_{m\_inf} \\ I_{adap\_inf} \\ I_{dep\_inf} \end{aligned} \right]$ (A 2)

where:

$c_{1}$, $c_{2}$, $c_{3}$ are arbitrary constants depending on the initial conditions

$\lambda_{1},\lambda_{2},\lambda_{3}$ are the eigenvalues of the coefficient matrix, with values:

$\lambda_{1}$ = ${-k}_{1}$, (∈ R^-^) (A 3)

$\lambda_{2}$ = $\frac{1}{2}\cdot\left[ \left( \frac{1}{\tau_{m}}-k_{2} \right)+\sqrt{\left( \frac{1}{\tau_{m}}-k_{2} \right)^{2}-4\cdot(-\frac{k_{2}}{\tau_{m}}+\frac{k_{adap}}{C_{m}})} \right]$ (A 4)

$\lambda_{3}$ = $\frac{1}{2}\cdot\left[ \left( \frac{1}{\tau_{m}}-k_{2} \right)-\sqrt{\left( \frac{1}{\tau_{m}}-k_{2} \right)^{2}-4\cdot(-\frac{k_{2}}{\tau_{m}}+\frac{k_{adap}}{C_{m}})} \right]$ (A 5)

$\boldsymbol{x}_{\mathbf{1}}$, $\boldsymbol{x}_{\mathbf{2}}$, $\boldsymbol{x}_{\mathbf{3}}$ are the eigenvectors associated to each eigenvalue

$V_{m\_inf}$, $I_{adap\_inf}$, $I_{dep\_inf}$ are the stationary solutions for each state variable.

For the membrane potential, the solution is (Hertäg et al., 2012):

$V_{m}\left( t \right)=c_{1}\cdot x_{1}^{(1)}\cdot e^{\lambda_{1}t}+c_{2}\cdot x_{2}^{(1)}\cdot e^{\lambda_{2}t}+c_{3}\cdot x_{3}^{(1)}\cdot e^{\lambda_{3}t}+V_{m\_inf}$ (A 6)

Specifically:

$V_{m\_inf}= E_{L}+\frac{-k_{2}\cdot\tau_{m}}{C_{m}\cdot k_{2}-k_{adap}\cdot\tau_{m}}\cdot(I_{e}+I_{stim})$ (A 7)

$x_{1}^{(1)}=\frac{{(k}_{1}-k_{2})\tau_{m}}{(k_{1}\tau_{m}-1){(k}_{2}-k_{1})C_{m}-k_{2}\tau_{m}}$, (A 8)

the first component of the eigenvector associated to the eigenvalue $\lambda_{1}$.

$x_{2}^{(1)}=x_{3}^{(1)}=1$, (A 9)

the first component of the eigenvectors associated to the eigenvalues $\lambda_{2}$ and $\lambda_{3}$, respectively

Considering that *k_1_* is real and positive, the dynamics of the solution depends on the discriminant:

$\Delta= \left( \frac{1}{\tau_{m}}-k_{2} \right)^{2}-4\cdot(-\frac{k_{2}}{\tau_{m}}+\frac{k_{adap}}{C_{m}})$ (A 10)

1. exponential and stable solution; the following conditions need to be verified:
   - $\boldsymbol{\Delta}>0$ ⇒ $\lambda_{1},\lambda_{2},\lambda_{3}$ ∈ R (the solution is exponential)
   - $\lambda_{1},\lambda_{2},\lambda_{3}$ *<* 0 (the solution is stable)

In addition, we need to verify that *V_m_inf_* ∝ *I_e_+I_stim_,* i.e. *V_m_inf_* is proportional to the total input current through a positive coefficient, to have a coherent value of steady state membrane potential.

These conditions result in the following constraints on parameters:

*⇒* $\left\{ \begin{aligned} k_{adap}<\frac{C_{m}}{4}{(k_{2}+\frac{1}{\tau_{m}})}^{2} \\ k_{adap}> \frac{C_{m}}{\tau_{m}}\cdot k_{2} AND k_{2}> \frac{1}{\tau_{m}} [\mathrm{stable}] \\ \end{aligned} \right.$ (A 11)

1. oscillatory solution; the following conditions need to be verified:
   - $\boldsymbol{\Delta}<0$ ⇒ $\lambda_{1},\lambda_{2},\lambda_{3}$ *∈ C* (the solution is oscillatory);
     - If *Re[*$\lambda_{1},\lambda_{2},\lambda_{3}$*] = 0* ⇒ the oscillations have null damping;
     - If *Re[*$\lambda_{1},\lambda_{2},\lambda_{3}$*] < 0* ⇒ the oscillations are damped and the solution is stable.

Analogously to the previous case, we need to verify that *V_m_inf_* ∝ *I_e_+I_stim_* through a positive coefficient.

The resulting constraints among parameters are:

*⇒* $\left\{ \begin{aligned} k_{adap}>\frac{C_{m}}{4}{(k_{2}+\frac{1}{\tau_{m}})}^{2} \\ k_{2}= \frac{1}{\tau_{m}} \left[ null damping \right] or k_{2}> \frac{1}{\tau_{m}} [stable] \left( \Rightarrow k_{2}>0 \right) \\ k_{adap}> \frac{C_{m}}{\tau_{m}}\cdot k_{2} (\mathrm{being} k_{2}>0 ) \end{aligned} \right.$ (A 12)

In this case, oscillations depend on the imaginary part of the eigenvalues ($\frac{\sqrt{\left| \Delta\right|}}{2}$) and thus have angular frequency $\omega= \frac{\sqrt{\left| \Delta\right|}}{2}$ and related frequency $f_{osc}= \frac{2\cdot\pi}{\omega}$.

The analytical solution was exploited in the E-GLIF optimization process to define the cost function and the parameter constraints.

During PyNEST simulations, the neuron model response over a membrane potential threshold was approximated to a spike. For neurophysiological realism, a spike was generated at time *t_spk_* depending on the escape rate function $\lambda(t)$ (Equ. 2) accounting for stochasticity and the refractory interval$\Delta t_{ref}$, if:

$\left\{ \begin{aligned} t_{spk}\notin\Delta t_{ref} \\ rnd<(1-e^{-\lambda(t_{spk})t_{spk}}) \end{aligned} \right.$ (A 13)

where *rnd* = random number in the interval [0, 1]

# E-GLIF optimization for cerebellar Golgi cells: parameter constraints

In order to obtain the expected neurophysiological behavior when simulating cerebellar GoCs, E-GLIF optimization took into account multiple parameter constraints. Specifically:

Nonlinear constraints:

Negative discriminant ($\Delta<0)$ to obtain an oscillatory membrane potential (as described in Appendix, I):

$k_{adap}>\frac{C_{m}}{4}{\cdot(k_{2}+\frac{1}{\tau_{m}})}^{2}$ (A 14)

Controlled *V_m_* oscillation frequency: 3 < *f_OSC_ < 8 Hz*, where *f_OSC_* is defined in Section 2.1.

Controlled amplitude of oscillations ($\left| V_{m\_osc} \right|$), during the intervals ${\Delta t}_{1}^{(zero\_stim)}$, ${\Delta t}_{2}^{(zero\_stim)}$, ${\Delta t}_{ss}^{(zero\_stim)}$ of the zero-current phase (*I_stim_* = 0 pA) and during the hyperpolarizing interval *hyp* (with *I_stim_ = inh*):

$\left| V_{m\_osc} \right|_{{\Delta t}_{1}^{(zero\_stim)},{\Delta t}_{2}^{(zero\_stim)},{\Delta t}_{ss}^{(zero\_stim)}}<100mV$ (A 15)

$\left| V_{m\_osc} \right|_{hyp}<200mV$ (A 16)

$V_{m}\left( 1.1\cdot t_{1\_des}^{(zero\_stim)} \right)> V_{th}$ to constraint the occurrence of the first spike event during the zero-current phase, so to trigger all the spike-reset-update mechanisms.

Linear constraints:

GoC show faster dynamics of the sodium ion current with respect to potassium one (accounted for by the current updates *A_1_* and *A_2_*):

$A_{1}>A_{2}>0$ pA (A 17)

Parameter bounds:

Not-damped *V_m_* oscillations (see Appendix, I):

$k_{2}= \frac{1}{\tau_{m}}$ (A 18)

After $\Delta t_{ref}$, negligible contribution of the depolarizing current *I_dep_*, during the zero-current phase (*I_stim_* = 0), to avoid interference with the neuron spontaneous activity, being *I_dep_* the depolarizing spike-triggered current, with decay *k_1_*:

$\frac{3}{mean(tonic\_freq)}< k_{1}< \frac{3}{\Delta t_{ref}}$ (A 19)

Realistic values of *I_dep_* and *I_adap_* based on the neurophysiological values of sodium and potassium ion currents (Solinas et al., 2007a):

$A_{1}$, $A_{2}$ **<** 500 pA (A 20)

Limited values of the endogenous current:

$0<I_{e}$ < 50 pA (A 21)
